# Cimetidine inhibits salivary gland tumor cell adhesion to neural cells and induces apoptosis by blocking NCAM expression

**DOI:** 10.1186/1471-2407-8-376

**Published:** 2008-12-18

**Authors:** Masakatsu Fukuda, Kaoru Kusama, Hideaki Sakashita

**Affiliations:** 1 Second Division of Oral and Maxillofacial Surgery, Department of Diagnostic and Therapeutic Sciences, Meikai University School of Dentistry, 1-1 Keyakidai, Sakado, Saitama 350-0283, Japan; 2 Division of Pathology, Department of Diagnostic and Therapeutic Sciences, Meikai University School of Dentistry, 1-1 Keyakidai, Sakado, Saitama 350-0283, Japan

## Abstract

**Background:**

Cimetidine, a histamine type-2 receptor antagonist, has been reported to inhibit the growth of glandular tumors such as colorectal cancer, however the mechanism of action underlying this effect is unknown. Adenoid cystic carcinoma is well known as a malignant salivary gland tumor which preferentially invades neural tissues. We demonstrated previously that human salivary gland tumor (HSG) cells spontaneously express neural cell adhesion molecule (NCAM), that HSG cell proliferation may be controlled via a homophilic (NCAM-NCAM) binding mechanism and that NCAM may be associated with perineural invasion by malignant salivary gland tumors. We further demonstrated that cimetidine inhibited NCAM expression and induced apoptosis in HSG cells. Here, we investigated the effects of cimetidine on growth and perineural/neural invasion of salivary gland tumor cells.

**Methods:**

In this study, we have examined the effect of cimetidine on cancer cell adhesion to neural cells *in vitro*, one of the critical steps of cancer invasion and metastasis. We have also used an *in vivo *carcinogenesis model to confirm the effect of cimetidine.

**Results:**

We have demonstrated for the first time that cimetidine can block the adhesion of HSG cells to neural cell monolayers and that it can also induce significant apoptosis in the tumor mass in a nude mouse model. We also demonstrated that these apoptotic effects of cimetidine might occur through down-regulation of the cell surface expression of NCAM on HSG cells. Cimetidine-mediated down-regulation of NCAM involved suppression of the nuclear translocation of NF-κB, a transcriptional activator of NCAM gene expression.

**Conclusion:**

These findings suggest that growth and perineural/neural invasion of salivary gland tumors can be blocked by administration of cimetidine via induction of apoptosis and in which NCAM plays a role.

## Background

Cimetidine, the first histamine type-2 receptor (H2R) antagonist to be used clinically, is commonly prescribed to treat gastro-esophageal reflux disease as well as gastric and duodenal ulcers [1]. It has been reported that cimetidine improves the survival of patients with malignant tumors [2,3], including gastric [4] and colorectal carcinomas [5]. Cimetidine has been shown to inhibit growth of gastrointestinal cancers via several mechanisms including enhancement of immune activity and inhibition of cancer cell proliferation [3]. Therefore cimetidine may act by enhancing the host immune response against tumor cells [6,7] or by blocking the cell growth-promoting activity of histamine [5,8-10]. Kobayashi *et al*. [11] reported that cimetidine inhibits colon adenocarcinoma cell adhesion to vascular endothelial cells and prevents metastasis by blocking E-selectin expression. We also demonstrated recently that cimetidine inhibited neural cell adhesion molecule (NCAM) expression and induced apoptosis in salivary gland tumor cells [12]. However, the exact mechanisms by which cimetidine suppresses the development of salivary gland tumors remain to be elucidated. Adenoid cystic carcinoma (ACC) is a well known and typical malignant salivary gland tumor. Facial paralysis due to perineural/neural invasion occurs so frequently that it is generally accepted as a hallmark of ACCs [13-15], and inhibition of perineural/neural invasion could be a strategy for arresting the development of ACC.

In this study, we have examined the effect of cimetidine on cancer cell adhesion to neural cells *in vitro*, one of the critical steps of cancer invasion and metastasis. We have also used an *in vivo *carcinogenesis model to confirm the effect of cimetidine. We demonstrated previously [16] that NCAM is spontaneously expressed in the human salivary gland tumor HSG cell line, derived from the submandibular salivary gland, and that HSG cell proliferation may be controlled via a homophilic (NCAM-NCAM) binding mechanism. We have also shown that NCAM may be involved in perineural/neural invasion by malignant salivary gland tumors [17]. Furthermore, it has been reported that NCAM expression is regulated by transcription factor κB (NF-κB) and that NF-κB activity is induced by homophilic NCAM binding [18,19]. NF-κB has key roles in inflammation, immune response, tumorigenesis and protection against apoptosis [20-22]. In most cell types, NF-κB remains bound to IκBα protein, and thereby inactive, in the cytoplasm [23,24]. After stimulation by various reagents, IκBα is rapidly phosphorylated by the IκB kinase (IKK) complex and degraded by the proteasome, allowing NF-κB to translocate to the nucleus and activate its target gene [21,25,26]. Here we found that cimetidine blocks not only salivary gland tumor cell adhesion to neural cells, but also tumor growth by inhibiting the NF-κB-mediated induction of NCAM. We also demonstrated that cimetidine can induce apoptosis in the inoculated tumor mass in a nude mouse model in which the salivary gland tumor cell line HSG was injected subcutaneously.

## Methods

### Reagents

For immunoblot analysis of NF-κB, MAb NF-κB p65/RelA (p65) antibody (Santa Cruz Biotechnology, CA, USA) was used as a primary antibody. Mouse anti-human NCAM monoclonal antibody (MAb NCAM antibody; CD56) was also purchased from Santa Cruz Biotechnology. MAb ICAM-1 was purchased from Medical & Biological Laboratories (Nagoya, Japan). Cimetidine was purchased from Sigma Chemical Co (MI, USA). H2R blockers were dissolved in PBS. Recombinant human TNF-α (R & D Systems, Inc., Minneapolis, MN) was used for the stimulation of HSG cells. MTT [3-(4,5-dimethylthiazol-2-yl)-2,5-diphenyl tetrazolium bromide] were obtained from Sigma.

### Cell culture

The HSG cell line, derived from a human submandibular salivary gland, was established by Shirasuna *et al*. [27] This cell line was maintained in RPMI 1640 medium supplemented with 10% heat-inactivated fetal bovine serum (FBS), 100 IU/mL penicillin and 100 mg/mL streptomycin and grown to confluency in 25 cm^2 ^culture flasks at 37°C in a humidified 5% CO_2 _incubator until required. Normal human neural progenitor (NHNP) cells and neural progenitor cell maintenance medium (NPMM) were purchased from Sanko Junyaku Co., Ltd. (Tokyo, Japan). NHNP cells were maintained with supplemented NPMM on 10 cm^2 ^polyethyleneimine (PEI) coated glass plates until they differentiated to neural cells.

### Co-culture of neural cells and HSG cells, and morphological observation of apoptotic cells

HSG cells (1 × 10^5 ^cells/ml) were added onto a semiconfluent monolayer culture of neural cells, incubated for 20 min at 37°C with rotation at 120 rpm, and washed extensively to exclude nonspecific cell attachment. The number of attached cells was counted directly under a microscope as reported previously [16]. The cells were then co-cultured for 24 h, after which they were further co-incubated with various concentrations of cimetidine (10^-8 ^to 10^-4 ^M) for 24 h. The co-cultured cells were washed extensively to exclude nonspecific cell attachment. The numbers of attached cells, and the morphological changes of each cell type, were observed directly under a confocal laser microscope. For antibody-mediated blocking of cell adhesion, the neural cells were incubated with antibody to either NCAM or ICAM-1 (final dilution of 1:200 for both antibodies) for 3 h at 37°C in humidified CO_2 _incubator, and then HSG cells were added.

### RNA extraction and real-time quantitative RT-PCR

Total RNA was extracted from monolayer HSG cells (1 × 10^6 ^cells/ml) treated with TNF-α (10 ng/ml) or various concentrations of cimetidine (10^-8 ^to 10^-4 ^M) by the acid-guanidinium-phenol-chloroform (AGPC) method reported previously [17]. To confirm the expression patterns of up-regulated or down-regulated NCAM and NF-κB genes after TNF-α or cimetidine treatment, real-time quantitative RT-PCR analyses were performed using a Bio-Rad iCycler system (Bio-Rad, Tokyo, Japan) and an iScript One-Step RT-PCR kit with SYBR Green I (Bio-Rad) according to the manufacturer's instructions. Briefly, the mRNAs were reverse-transcribed into cDNAs at 50°C for 10 min and reverse transcriptase was inactivated at 95°C for 5 min. PCR cycling and detection were followed by running for 45 cycles at 95°C for 10 s and 56°C for 30 s. PCR primers were designed and synthesized by Sigma-Aldrich, Inc. (Ishikari, Japan) with consideration of the special design criteria for real-time PCR primers. The following primer sequences were used in the PCR reactions: NCAM forward, GAA TGC CAC CGC CAA CCT C; NCAM reverse, GTC TTC CTC TTG CTC TAT CTG TTC C; NF-κB forward, AGG CGA GAG GAG CAC AGA TAC; NF-κB reverse, CGG CAG TCC TTT CCT ACA AGC; GAPDH forward, CAG CCT CAA GAT CAT CAG CA; GAPDH reverse, ACA GTC TTC TGG GTG GCA GT. Each sample was amplified in triplicate and the corresponding no-RT mRNA sample was included as a negative control. The relative mRNA level of each sample for each gene was normalized to the mRNA level of GAPDH, a housekeeping gene. The results were analyzed with the Bio-Rad iCycler Software 3.0 and Microsoft Excel 97 and presented as fold induction compared with the quantity of GAPDH mRNA (set at 1). The specificities of PCR products were analyzed by melting curve data and agarose gel electrophoresis to determine product size and to confirm that no by-products were formed.

### Protein extraction

To examine RelA translocation to the nucleus, we used a subcellular proteome extraction kit (S-PEK; Calbiochem, Darmstadt, Germany) according to the manufacturer's instructions to extract cytoplasm, cell membrane and nucleus fractions of HSG cells. Cells were treated with 10 ng/ml of TNF-α and/or 10^-4 ^M cimetidine, pelleted (5 × 10^6 ^cells), washed twice, resuspended in 1 ml of ice-cold Extraction I containing 5 μl of protease inhibitor mixture, and then incubated for 10 min at 4°C with gentle agitation. The suspension was centrifuged at 1000 × *g *at 4°C for 10 min. The supernatant was used as the cytoplasm fraction; the pellet was resuspended in 1 ml of ice-cold Extraction II containing 5 μl of protease inhibitor mixture and incubated for 30 min at 4°C. It was then centrifuged at 6000 × *g *at 4°C for 10 min, and the supernatant was used as the cell membrane fraction; the pellet was resuspended in 500 μl of ice-cold Extraction III containing 5 μl of protease inhibitor mixture and 1.5 μl of Benzonase^® ^and incubated for 10 min at 4°C with gentle agitation. It was then centrifuged at 7000 × *g *at 4°C for 10 min, and the supernatant was used as the nucleus fraction. Each sample was subjected to immunoblot analysis.

### Immunoblot analysis

For the detection of RelA protein by gel electrophoresis, 10 μg protein samples were mixed with an equal volume of SDS-PAGE sample buffer and boiled for 5 min. Each sample was loaded into a single lane and separated on a polyacrylamide gel of appropriate percentage. The proteins were then electroblotted onto nitrocellulose membranes. Subsequent immunoblot analysis was carried out according to the method reported previously [17]. Filters were scanned and computer-generated images were analyzed with the National Institutes of Health IMAGE program to obtain densitometric values. For each series of samples (cytoplasm, cell membrane and nucleus), the relative density of each image was calculated and expressed as a percentage of the value (arbitrarily set at 100) indicated by an asterisk.

### Transfection of HSG cells with plasmids and luciferase reporter assay

HSG cells (1 × 10^5 ^cells/ml) were cultured for 12 h in 24-well culture plates containing RPMI1640 supplemented with 10% FBS. pTKκB2luc, a thymidine kinase (TK) luciferase construct containing five copies of the κB motif from the CXCL10/IP-10 gene, was kindly provided by Professor Y. Ohmori [28]. Cells were transiently transfected with pTKκB2luc and pRL-TK reference *Renilla *luciferase plasmids (Promega, Madison, WI, USA) using FuGene transfection reagents (Roche, Nutley, NJ, USA), according to the manufacturer's instructions. At 24 h after transfection, the cells were treated with TNF-α for various periods (0, 1, 4, 8 and 24 h). Firefly and *Renilla *luciferase activities were assayed using reagents provided by Promega, according to the manufacturer's instructions. For standardization of transfection efficiencies, the luciferase activity from pTKκB2luc was normalized to the *Renilla *luciferase activity. The pGL3 control luciferase plasmid was purchased from Promega.

### RNA-mediated interference

Small interfering RNAs (siRNAs) specific for human NCAM, NF-κB and scrambled (control) were synthesized by Sigma-Aldrich, Inc. (Ishikari, Japan). The sense and antisense strand sequences of the oligonucleotides were as follows: NCAM siRNA sense, GCA AUA UCA AGA UCU ACA ATT; antisense, UUG UAG AUC UUG AUA UUG CTT; control siRNA sense, AAU CAC AAU UGC GCA AUA ATT; antisense, UUA UUG CGC AAU UGU GAU UTT; NF-κB siRNA sense, GGG UAU AGC UUC CCA CAC UTT; antisense, AGU GUG GGA AGC UAU ACC CTT; control siRNA sense, AGU ACU CUC GUA CGC UCG ATT; antisense, UCA UGA GAG CAU GCG AGC UTT. Before transfection, FuGene 6 transfection reagent was mixed with 100 nM NCAM, 100 nM NF-κB or 100 nM control siRNA (3:3.4 μl) in serum-free medium, to a total volume of 500 μl and incubated for 30 min at room temperature. For NF-κB knockdown, HSG cells (1 × 10^5 ^cells/ml) were rinsed with serum-free medium and transfected in 24-well plates with either a NF-κB siRNA duplex or a control siRNA, using FuGene 6 transfection reagents for 48 h at 37°C. Cells were treated with TNF-α for various periods (0–24 h) and subjected to a Real-Time Quantitative RT-PCR. For NCAM knockdown, HSG cells (1 × 10^5 ^cells/ml) were rinsed with serum-free medium and transfected in 24-well plates with an NF-κB-dependent luciferase reporter plasmid and either a NCAM siRNA duplex or a control siRNA for 48 h at 37°C. Cells were treated with TNF-α for 4 h and subjected to a luciferase reporter assay and immunoblot analysis.

### Nude mouse model of HSG tumor mass

All of the experimental procedures were performed with the approval of the Animal Experimentation Committee of the Meikai University School of Dentistry. Specific pathogen-free athymic BALB/c female mice, 3–4 weeks of age, were kept under sterile conditions in a laminar flow room in cages with filter bonnets and fed a sterilized mouse diet and water. The mice were anesthetized by inhalation of diethyl ether. HSG cells (1 × 10^6 ^cells) in 100 μl of PBS were injected subcutaneously into the back of each mouse with a 27-gauge needle. Tumor size was measured daily with calipers, and the HSG tumor masses in all mice, movable and elastic-hard, grew to approximately 10 mm in diameter by three weeks after HSG cell inoculation. To examine the effects of H2R antagonists, the mice were treated with 10^-4 ^or 10^-2 ^M/day cimetidine or saline (control) by intratumoral injection for seven consecutive days after the HSG tumor mass had reached approximately 10 mm in diameter. Subsequently, cimetidine or saline (control) was administered every other day for an additional 5 weeks (a total of 9 weeks after HSG cell implantation).

### Quantification and apoptosis detection of tumor mass in nude mice

At nine weeks after HSG cell injection, the animals were sacrificed and the status of the tumor mass was evaluated quantitatively (Table 1). The tumor volume (V, mm^3^) was calculated as 0.5 × L × W^2^, where L = length (mm) and W = width (mm), respectively. The percentage of tumor growth inhibition was expressed as the mean (± SD) tumor volumes (calculated in each group of ten mice) relative to the volume of tumors injected with control saline. The back skin was excised and cut into 2–3-mm thick slices. Then, a formalin-fixed, paraffin-embedded specimen was obtained for subsequent H&E staining and apoptosis analysis. Terminal deoxynucleotidyl transferase (TDT) dUTP nick end labelling (TUNEL) method was performed for evaluation of apoptotic cells using an *in situ *apoptosis detection kit (Takara, Shiga, Japan). Briefly, the deparaffinized sections were treated with 20 μg/ml proteinase K for 15 min and immersed in absolute methanol containing 0.3% H_2_O_2 _for 10 min at room temperature to block endogenous peroxidase activity. After washing in PBS, sections were incubated with TDT enzyme in a humidified chamber at 37°C for 1 h. After washing with PBS, the slides were incubated with diluted streptavidin-peroxidase (1:1000) for 30 min at room temperature. Positive cells were visualized using a diaminobenzidine substrate and counterstained with hematoxylin. TUNEL positive cells showed dark gray staining of the nucleus suggestive of internucleosomal DNA cleavage.

**Table 1:** Effect of cimetidine at 9 weeks after subcutaneous inoculation of HSG cells in nude mice

	Control (saline)	Cimetidine (M/day)	
		
		10^-4^	10^-2^
Injected 1 × 10^6 ^cells	9/10 (90%)	4/10 (40%)^a^	0/10 (0%)^b^
Mean Tumor Volume, mm^3 ^± SD (% control)	600.9 ± 12.97 (100%)	58.35 ± 2.67 (9.71%)^a^	0 (0%)^b^

A mouse in the control group (1/10) died unexpectedly. Tumor volume was calculated as width^2 ^× length × 0.5.

^a ^*P *< 0.05 (compared with control).

^b ^*P *< 0.001.

### Statistical analysis

The correlations between the results of experimental treatments were evaluated by the two-tailed Student's *t *test. Differences in probability values (*p*-values) of less than 0.05 were considered statistically significant in all analyses. All analyses were performed with StatView statistical software (version 5.0; SAS Institute Inc., Cary, NC).

### Ethical considerations

The study was approved by the Research Ethics Committee of the Meikai University School of Dentistry, Saitama, Japan (reference number: A0801).

## Results

### Suppression of tumor cell adhesion to neural cells by cimetidine

**Figure 1:**
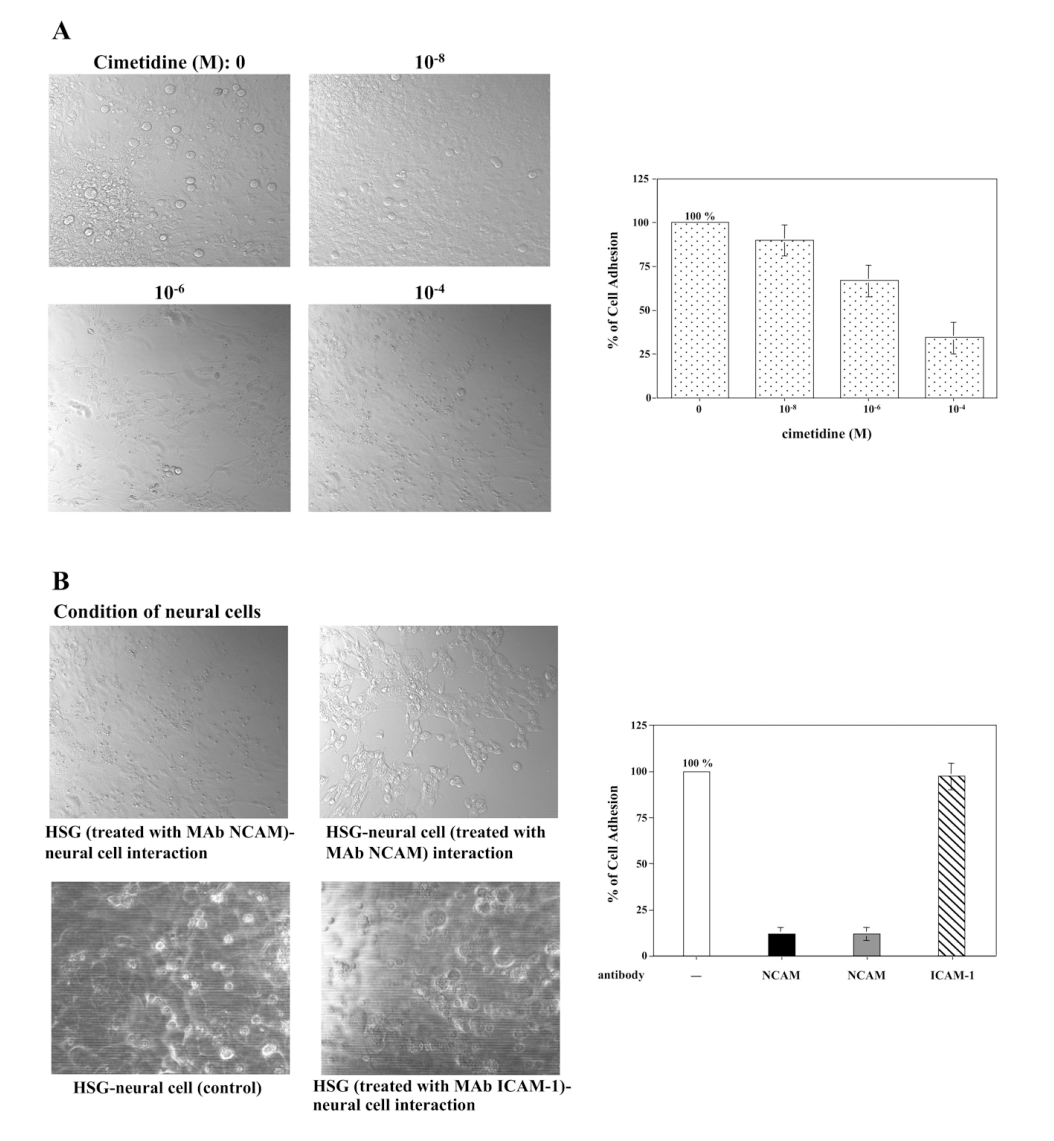
**Inhibition of HSG tumor cell adhesion to neural cells**. *A*, various concentrations of cimetidine were added, and the interaction of HSG cells with neural cells was examined. Phase-contrast microscopic pictures of representative experiments are shown. The number of HSG cells adhering to the neural cell monolayer was counted and is shown in the bar graph. *B*, NCAM antibody was added and the interaction of HSG cells with neural cells was examined. Phase-contrast microscopic pictures of representative experiments are shown. The number of HSG cells adhering to the neural cell monolayer was counted and is shown in the bar graph. The label 'HSG (treated with MAb NCAM)-neural cell interaction' and the black column in the graph indicate the number of HSG cells adhering to the neural cells when the HSG cells were preincubated with the MAb NCAM. The label 'HSG-neural cell (treated with MAb NCAM) interaction' and the gray column in the graph indicate the number of HSG cells adhering to the neural cells when the neural cells were preincubated with the MAb NCAM. ICAM-1 antibody was also added, and the results are shown as a label 'HSG (treated with MAb ICAM-1)-neural cell interaction' and in the diagonally shaded column (n = 3 experiments; means ± SD).

To examine the effects of cimetidine on HSG cell adhesion to neural cells, a monolayer cell adhesion assay was carried out. As shown in Fig. 1A, HSG cells adhered strongly to neural cells. Using this model, we investigated the effects of various H2R antagonists at a range of non-cytotoxic concentrations (from 10^-8 ^to 10^-4 ^M [8,9]; also confirmed by MTT assay in a previous study [12]). Cultured neural cells were pretreated with cimetidine or other H2R antagonists for 24 h before the addition of HSG cells. After HSG cells were added, the cell adhesion assay was performed. As shown in Fig. 1A, adhesion of HSG cells to neural cells was inhibited by cimetidine in a dose-dependent manner. However, the other H2R antagonists, famotidine and ranitidine, had no inhibitory effect (data not shown). Aithough HSG cells and neural cells were further co-incubated with various concentrations of cimetidine (10^-8 ^to 10^-4 ^M) for 24 h, morphological change to apoptotic cells was not observed in neural cells. However, HSG cells obviously underwent apoptosis. This finding has shown in a previous study [12]. To confirm that HSG adhesion to neural cells was a result of a cognate interaction between NCAM (on HSG) and NCAM (on neural cells), specific antibodies were added to block these molecules before the monolayer cell adhesion assay. Preincubation of HSG cells with an MAb NCAM abolished their ability to adhere to neural cells (Fig. 1B). Similarly, when neural cells were preincubated with the MAb NCAM, HSG adhesion to neural cells was also blocked (Fig. 1B). In contrast, pre-incubation of HSG cells with anti-ICAM-1 antibody did not significantly reduce cell adhesion (Fig. 1B, *photo and graph*). These findings indicate that homophilic NCAM binding is the primary mediator of cell adhesion between HSG cells and neural cells in this assay system.

### Effects of cimetidine on NCAM and NF-κB gene expression

**Figure 2:**
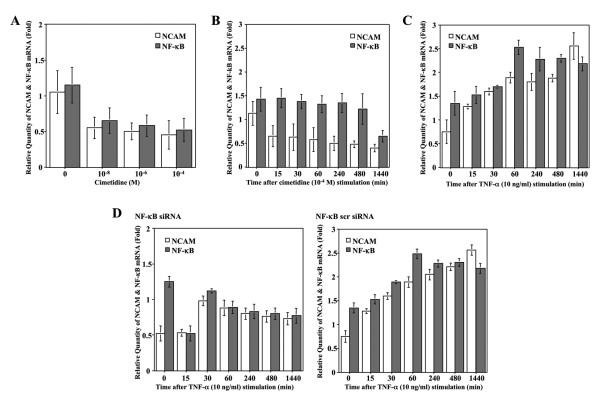
**Effects of cimetidine on NCAM and NF-κB gene expression**. *A*, the relative quantities of both NCAM and NF-κB mRNA were suddenly decreased by treatment with 10^-8 ^M cimetidine for 24 h, and then kept decreasing slightly by treatment with the higher doses of it. *B*, the relative quantity of NCAM mRNA was rapidly decreased in the first 15 min, and then continued to decrease in a time-dependent manner up to 24 h. However, the relative quantity of NF-κB mRNA showed almost no variation up to 8 h, and after that decreased at 24 h. *C*, initially, the relative quantity of NF-κB mRNA was constitutively induced in HSG cells and was gradually increased upon stimulation with TNF-α, an effect that reached a peak at 60 min and then decreased up to 24 h in a time-dependent fashion. With an increasing quantity of NF-κB mRNA, the relative quantity of NCAM mRNA was gradually increased upon stimulation with TNF-α up to 24 h in a time-dependent manner. *D*, NF-κB siRNA greatly reduced NCAM mRNA expression in HSG cells, in comparison with transfection using NF-κB scrambled siRNA. Each column and bar represents the mean ± S.E.M. of three independent experiments.

To investigate the effect of cimetidine on NCAM and NF-κB mRNA levels, real-time quantitative RT-PCR analysis was carried out. Total RNA purified from HSG cultures treated with various concentrations of cimetidine at non-cytotoxic concentrations (from 10^-8 ^to 10^-4 ^M) was quantitated by real-time RT-PCR using specifically designed primer pairs. Surprisingly, the relative quantities of both NCAM and NF-κB mRNA rapidly decreased after treatment with 10^-8 ^M cimetidine for 24 h, and then continued to decrease further at higher doses of cimetidine (Fig. 2A). As a second step, we performed a time-course analysis on NCAM and NF-κB mRNA expression in HSG cells treated with 10^-4 ^M cimetidine (Fig. 2B). The relative quantity of NCAM mRNA rapidly decreased in the first 15 min, and then gradually decreased in a time-dependent manner. However, the relative quantity of NF-κB mRNA showed almost no variation up to 8 h, but had decreased after 24 h. Thus, while the rate of induction of NCAM mRNA declined rapidly, induction of NF-κB mRNA only declined slowly. These findings indicate that cimetidine time- and dose-dependently down-regulates the expression of NCAM mRNA and also reduces NF-κB mRNA expression in HSG cells in a dose-dependent fashion. As a control experiment, we performed a time-course analysis of NCAM and NF-κB mRNA expression in HSG cells treated with 10 ng/ml of TNF-α (Fig. 2C). At first, the relative quantity of NF-κB mRNA was constitutively induced in HSG cells and was gradually increased upon stimulation with TNF-α, an effect that reached a peak at 60 min and then time-dependently decreased up to 24 h. With an increasing quantity of NF-κB mRNA, the relative quantity of NCAM mRNA was gradually increased upon stimulation with TNF-α up to 24 h in a time-dependent manner. These results strongly suggest that NCAM mRNA is induced by the activation of NF-κB and may play a critical role in proliferative activity of HSG cells. To confirm whether NF-κB activation indeed increased NCAM mRNA in HSG cells, we performed a siRNA approach to reduce the expression of NF-κB and determined the effects on the basal and TNF-α-induced activity of NF-κB. As shown in Fig 2D, NF-κB knockdown by NF-κB siRNA greatly reduced NCAM mRNA expression in HSG cells, in comparison with transfection using NF-κB scrambled siRNA. These data indicate that NCAM mRNA is indeed induced by the activation of NF-κB in HSG cells.

### Cimetidine inhibits TNF-α-mediated NF-κB activation in HSG cells

**Figure 3:**
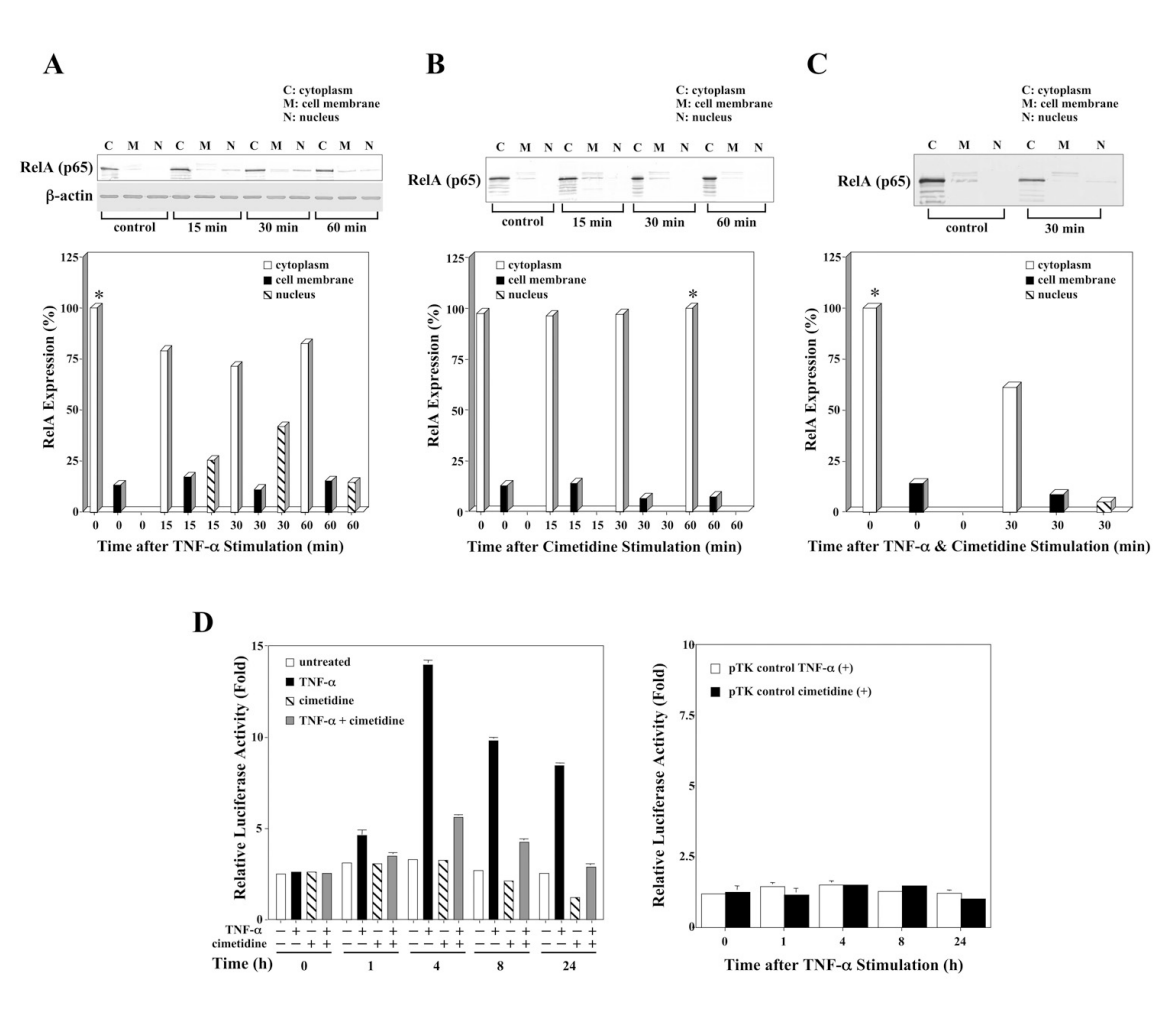
**Effect of cimetidine on TNF-α-mediated NF-κB activation in HSG cells.***A*, RelA protein was localized mainly in the cytoplasm of HSG cells and transported to the nucleus upon stimulation by TNF-α; it reached a peak at 30 min and then decreased at 60 min. *B*, the intensity of RelA expression in the HSG cell membrane was decreased by treatment with cimetidine in a time-dependent manner, whereas the intensity of RelA in the cytoplasm after treatment with 10^-4^ M cimetidine gradually increased up to 60 min. *C*, RelA transport was detected in the nucleus of HSG cells stimulated with 10 ng/ml of TNF-α and 10^-4^ M cimetidine for 30 min. In comparison with treatment with TNF-α alone, however, the intensity of RelA expression was markedly inhibited. *D, left*, maximum κB-dependent transcription was observed with 10 ng/ml of TNF-α at 4 h, which induced a 5-fold increase in luciferase activity compared with cells not exposed to TNF-α, and did not further increase with time. However, luciferase activities in HSG cells were time-dependently decreased in response to 10-4 M cimetidine from 4 h after cimetidine treatment. Half-maximal inhibition of luciferase activity was detected at 24 h after stimulation with 10^-4^ M cimetidine. Induction of luciferase activity by TNF-α was inhibited by cimetidine in a time-dependent fashion. *D, right*, the increase or decrease in luciferase activity was completely dependent on the presence of κB sites, since the control plasmid lacking the κB elements did not respond to TNF-α or cimetidine. Relative luciferase activities are shown as -fold induction compared with the activity of cimetidine-treated samples at 24 h *(D, left)* or untreated *(D, right)* samples. Each column and bar represents the mean ± S.E.M. of three independent experiments.

To examine how RelA expression is regulated in HSG cells upon stimulation with TNF-α, immunoblot analysis was carried out followed by densitometric analysis. RelA protein was localized in the cytoplasm of HSG cells and was transported to the nucleus upon stimulation with TNF-α, an effect that reached a peak at 30 min and then decreased at 60 min (Fig. 3A). When the time course of RelA protein expression was analyzed up to 24 h, nuclear translocation of RelA was further decreased (data not shown). Regulation of RelA expression in HSG cells after stimulation with cimetidine was also analyzed. RelA was primarily localized in the cytoplasm with small quantities also detected in the cell membrane. The intensity of RelA expression in HSG cell membranes was decreased in a time-dependent manner by treatment with 10^-4 ^M cimetidine up to 60 min, whereas the intensity of RelA expression in cytoplasm of HSG cells gradually increased up to the same period (Fig. 3B). Interestingly, although the quantity of NCAM mRNA was time-dependently down-regulated in HSG cells treated with 10^-4 ^M cimetidine, real-time quantitative RT-PCR showed that the relative amount of NF-κB mRNA was not markedly down-regulated early after treatment (Fig. 2B). Taken together, these results show that cimetidine treatment of HSG cells resulted in complete inhibition of nuclear translocation of NF-κB, even though the relative quantity of NF-κB mRNA is sufficiently observed. Thus down-regulation of NCAM is induced via suppression of NF-κB activity by cimetidine. This effect may play a critical role in cimetidine-induced apoptosis of HSG cells.

We also examined the effect of cimetidine on TNF-α-induced nuclear translocation of RelA. RelA transport to the nucleus was detectable in HSG cells stimulated with 10 ng/ml of TNF-α and 10^-4 ^M cimetidine for 30 min (Fig. 3C). However, transport was markedly inhibited when compared with treatment with TNF-α alone. In contrast, the other H2R antagonists failed to block the nuclear translocation of RelA induced by TNF-α (data not shown). To investigate the effect of TNF-α on NF-κB-dependent transcriptional activity in HSG cells, a luciferase reporter assay was performed. TNF-α caused strong induction of luciferase activity (Fig. 3D, left). Maximum NF-κB-dependent transcription was observed at 4 h, with a 5-fold increase in luciferase activity compared with cells not exposed to TNF-α, and no further increase with time was observed. However, constitutive NF-κB activity was not observed in HSG cells. The increase in luciferase activity was completely dependent on the presence of κB sites, since the control plasmid lacking the κB elements did not respond to TNF-α (Fig. 3D, right). Furthermore, luciferase activities in HSG cells were time-dependently decreased in response to 10^-4 ^M cimetidine from 4 h after cimetidine stimulation (Fig. 3D, left). Half-maximal inhibition of luciferase activity was detected at 24 h after stimulation with 10^-4 ^M cimetidine.

To test whether cimetidine can suppress induction of luciferase activity after TNF-α stimulation, a luciferase reporter assay was also carried out. Induction of luciferase activity by TNF-α was inhibited by cimetidine in a time-dependent fashion (Fig. 3D, left). The decrease in luciferase activity was completely dependent on the presence of κB sites, since the control plasmid lacking the κB elements did not respond to cimetidine (Fig. 3D, right). Together, these data indicate that cimetidine has a specific and potent inhibitory effect on NF-κB-dependent transactivation.

### NCAM is a regulator for NF-κB activation in HSG cells

**Figure 4:**
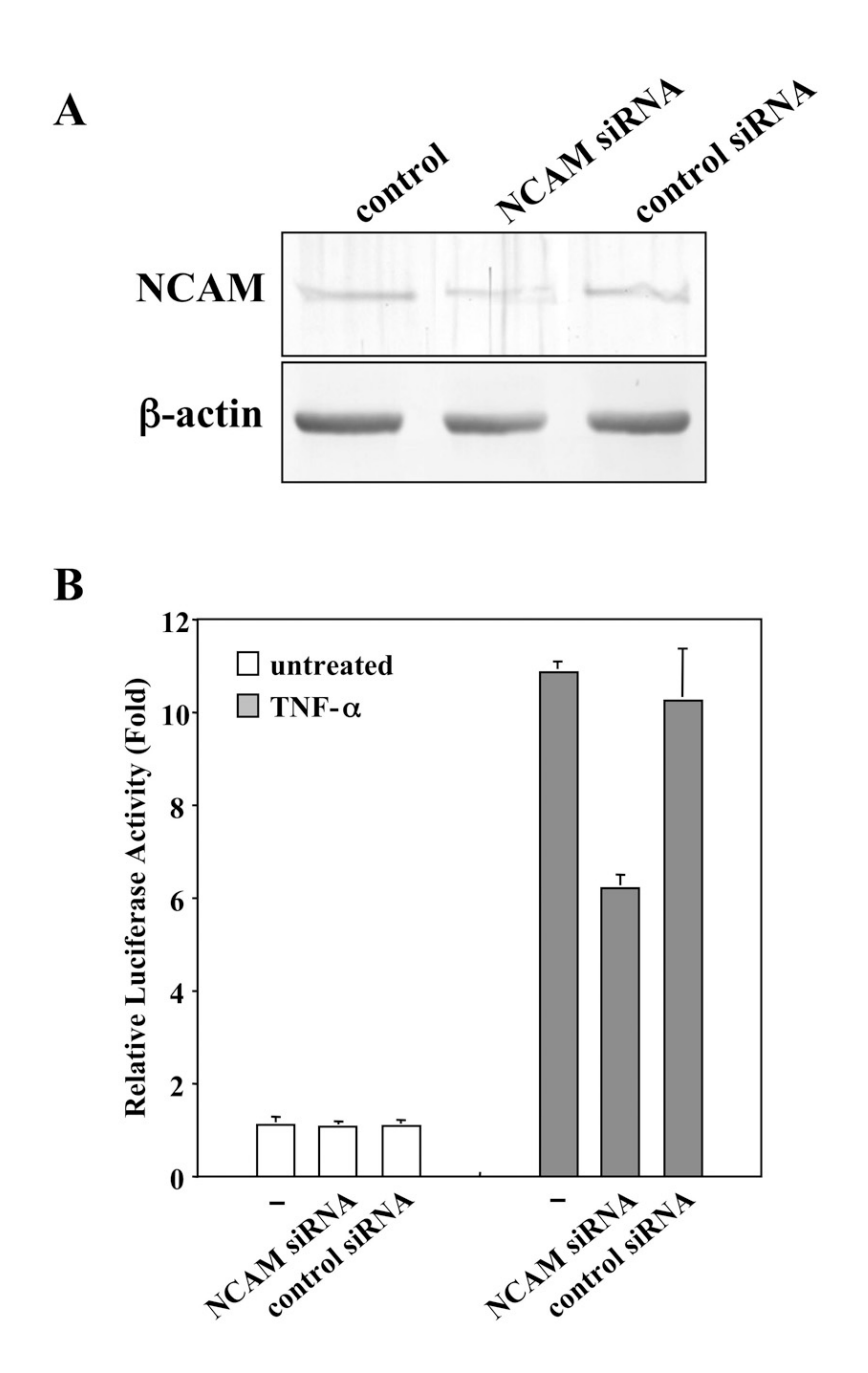
**NCAM siRNA-mediated interference**. *A*, endogenous NCAM protein was markedly reduced by NCAM siRNA. *B*, NCAM siRNA greatly reduced activation of NF-κB by TNF-α in HSG cells, in comparison with NCAM scrambled siRNA transfection. NCAM siRNA had no effect on constitutive NF-κB activity in HSG cells. The relative luciferase activities are shown as -fold induction compared with the activity of untreated samples. Each column and bar represents the mean ± S.E.M. of three independent experiments.

To determine whether endogenous NCAM indeed functions as a regulator for NF-κB activation by TNF-α in HSG cells, we used a siRNA approach to reduce the expression of NCAM and determined the effects on the basal and TNF-α-induced activity of NF-κB. As expected, the NCAM protein was markedly reduced by NCAM siRNA (Fig. 4A). We then assessed the effect of NCAM siRNA on NF-κB-dependent transcriptional activity by TNF-α for 4 h. As shown in Fig 4B, NCAM knockdown by NCAM siRNA greatly reduced NF-κB activation by TNF-α in HSG cells, in comparison with transfection using NCAM scrambled siRNA. However, NCAM siRNA had no effect on constitutive NF-κB activity in HSG cells. These data indicate that NCAM functions as a regulator for NF-κB activation induced by TNF-α in HSG cells.

### Suppression of tumor growth and induction of apoptosis in the tumor mass in nude mice by cimetidine

**Figure 5:**
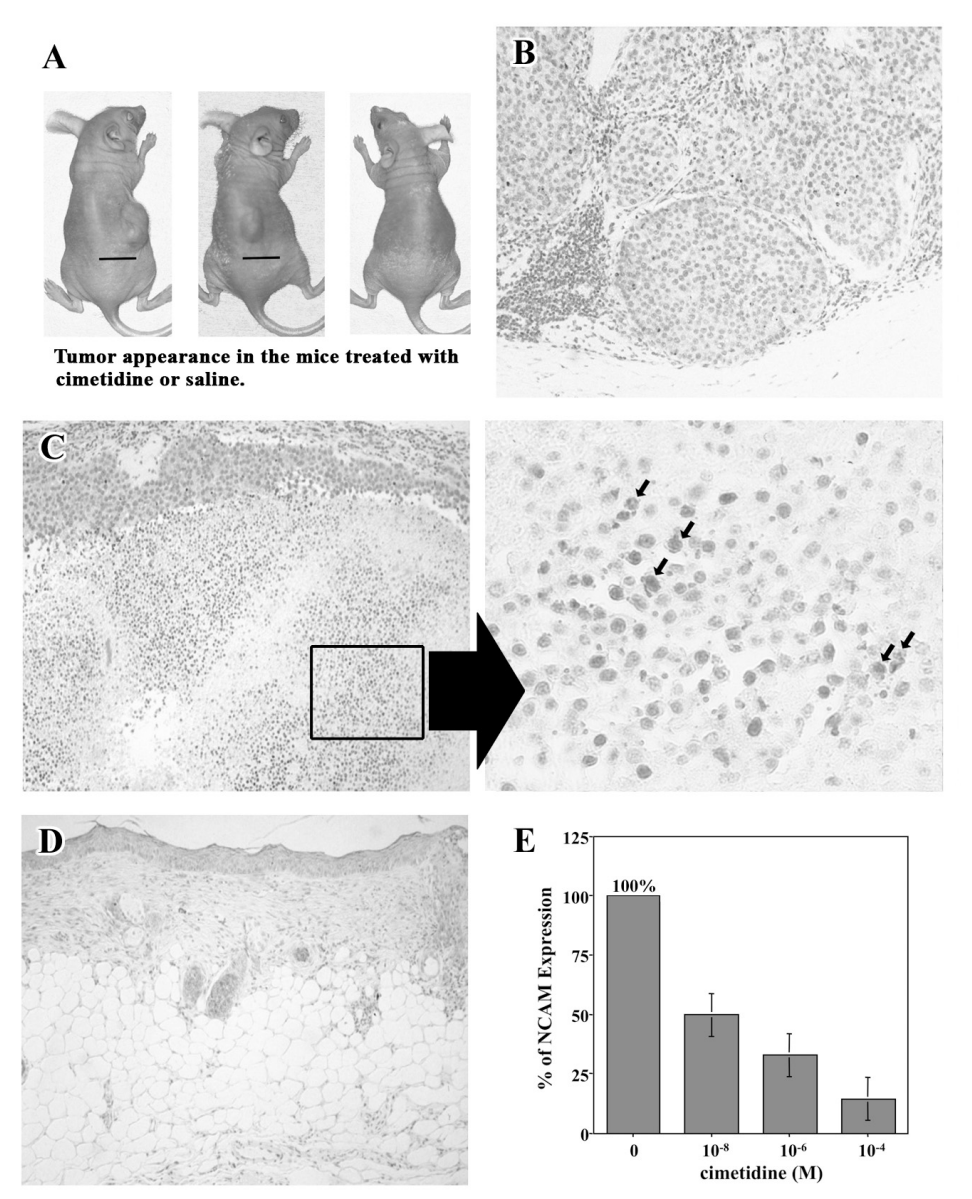
**Induction of apoptosis in the tumor mass in nude mice by cimetidine**. *A*, tumor appearance in mice treated with cimetidine or saline. Bar, 10 mm. *A, left*, saline was administered to the mice. *A, middle*, 10^-4 ^M cimetidine was administered to the mice. Regression of HSG tumor mass was observed. *A, right*, 10^-2 ^M cimetidine was administered to the mice. The HSG tumor mass was completely resolved. *B*, histopathological findings revealed that the tumor mass was composed of several nodular tumors. The small nodes were composed of large round tumor cell cords clearly demarcated from the stroma. Inflammatory small round cell infiltration was found in the stroma. *C*, at 10^-4 ^M cimetidine, apoptotic cell death was markedly observed by TUNEL in 4 of 10 mice (40%) in which HSG tumor masses had formed. Significant morphologic changes, including shrinkage of cytoplasm, fragmented nuclei and release of apoptotic vesicles in HSG tumor mass, were also indicated by arrows (*right*). *D*, at the highest dose of cimetidine (daily doses of 10^-2 ^M), the HSG tumor mass was completely resolved when 1 × 10^6 ^HSG cells were injected subcutaneously. *E*, the expression of NCAM in HSG tumor mass was decreased by cimetidine in a dose-dependent fashion. Each column and bar represents the mean ± S.E.M. of three independent experiments.

We then examined the effects of cimetidine on tumor development *in vivo *in a nude mouse model. Mice were inoculated with HSG (1 × 10^6 ^cells) subcutaneously (Fig. 5A), and the effects of cimetidine on the extent of the HSG tumor mass were evaluated. Nine weeks after the injection of HSG cells, mice were sacrificed and the status of the tumor mass was examined histopathologically. As demonstrated in Table 1, cimetidine not only prevented the growth of the HSG tumor mass, but also induced a dose-dependent reduction in tumor volume. One mouse in the control group (1/10) died unexpectedly. As shown in Fig. 5B, histopathological findings revealed that the tumor mass was composed of several nodular tumors. The small nodes were composed of large round tumor cell cords clearly demarcated from the stroma. Inflammatory small round cell infiltration was found in the stroma. At 10^-4 ^M cimetidine, HSG tumor mass in 6 of 10 mice (60%) was resolved. Although remaining tumor cells were identified in 4 of 10 mice (40%) in which an HSG tumor mass had formed, there was clear regression of HSG tumor mass (Table 1) with evidence of extensive apoptotic cell death as observed by TUNEL in HSG tumors treated with 10^-4 ^M cimetidine (Fig. 5C). The significant morphologic changes, including shrinkage of cytoplasm, fragmented nuclei and release of apoptotic vesicles in HSG tumor mass, were also observed by the microscopic analysis (Fig. 5C, right). At the highest dose of cimetidine (daily doses of 10^-2 ^M), the HSG tumor mass was completely resolved when 1 × 10^6 ^HSG cells were injected subcutaneously (none of the ten mice had detectable tumor mass; Table 1 and Fig. 5D). Famotidine or ranitidine at doses equivalent to that of cimetidine had no inhibitory effect on the growth of the HSG tumor mass (data not shown). In a repeat experiment using a greater number of HSG cells (1 × 10^7^) for inoculation, cimetidine demonstrated similar effects on the prevention of tumor growth (data not shown). Finally, immunohistochemical examination revealed that the expression of NCAM in HSG tumor mass was decreased by cimetidine in a dose-dependent fashion (Fig. 5E). These findings indicate that cimetidine effectively reduces the expression of NCAM, and ultimately induces apoptosis to HSG tumor masses in nude mice.

## Discussion

Cimetidine, the most studied H2R antagonist, has been shown to possess anti-tumor activity against colon, gastric and kidney cancers, and melanomas [5,8-10]. A recent study suggested that this action of cimetidine may be mediated by three very different effects: a direct inhibitory effect on tumor growth by blocking the cell growth-promoting activity of histamine via activation of H2 receptors, and an indirect effect involving inhibition of tumor-associated angiogenesis; an immunomodulatory effect through enhancement of the host's immune response to tumor cells; and an inhibitory effect on cancer cell migration and adhesion to endothelial cells, thus inhibiting tumor neo-angiogenesis and metastasis [29]. In this study, we investigated the NCAM-associated effect of cimetidine on tumor growth and perineural/neural invasion in salivary gland tumors using an *in vitro *cell culture system and an *in vivo *nude mice cancer model. These experiments clearly demonstrated that cimetidine effectively down-regulates the induction of NCAM by inhibiting the transactivation of NF-κB, subsequently blocks HSG adhesion to neural cells, and ultimately induces apoptosis in HSG cells and prevents the growth of HSG tumor masses in nude mice.

NCAM is a membrane glycoprotein receptor of the immunoglobulin supergene family that mediates cell-to-cell adhesion via homophilic binding to other NCAM molecules and cell-to-substrate adhesion via heterophilic binding (NCAM binding to another ligand or counter-receptor) [30]. NCAM plays an important role in perineural invasion in various neoplasms, such as bile duct cancer, gallbladder carcinoma, melanoma and adenoid cystic carcinoma of the head and neck [13-15,31-33]. In a previous study [16], we reported that adenoid cystic carcinoma is positive for NCAM and that adhesion of the tumor cell line HSG to neural cells depends on NCAM expression on the cell surface of both HSG and neural cells. In addition, our previous study [12] showed that the levels of NCAM mRNA and protein in HSG cells were decreased by cimetidine in a dose-dependent manner. Furthermore, cimetidine induced significant apoptosis in HSG cells by activation of caspases via the DNA-damage signal mediated through mitochondria. As mentioned above, several of the actions of cimetidine, such as the direct growth-inhibitory effects on certain cancer cell lines [1,10] and direct stimulatory effects on lymphocyte function [5-7] were unexpected. Some studies have also indicated that cimetidine may have antioxidant activity [34-36], and antioxidants have been shown to block the NF-κB activation cascade [37]. In addition, it has also been reported that NCAM expression is regulated by NF-κB [18], that NF-κB activity is induced by NCAM [19], and that NF-κB activates the expression of a number of genes at the transcription level [21,25,26]. In other words, these observations imply that homophilic NCAM binding can increase NF-κB activity and that NF-κB regulates NCAM expression.

In this study, the results of real-time quantitative RT-PCR indicated that the activated NF-κB induced NCAM expression, and the NCAM knockdown analysis suggested that NCAM regulates NF-κB activation induced by TNF-α in HSG cells. It has already been reported that TNF-α induces NF-κB activation via a common pathway based on the phosphorylation-induced degradation of IκBs [25]. In the present study, TNF-α was also used to activate NF-κB in HSG cells to mimic the local inflammatory response in the metastasized region, and some tumor cells were reported to produce TNF-α [25]. We found that NF-κB activation in response to TNF-α was blocked by cimetidine, suggesting that cimetidine interferes with an early common signal in the TNF-α signal transduction cascade. Cimetidine treatment also inhibited translocation of NF-κB into the nucleus. Thus two separate of lines of evidence allow us to conclude that cimetidine is a potent and specific inhibitor of NF-κB activation in HSG cells: (a) cimetidine inhibited TNF-α-induced nuclear transactivation of NF-κB, and (b) cimetidine suppressed NF-κB-dependent transcription. Indeed, NF-κB controls genes that code for anti-apoptotic proteins, some acting at the mitochondrial level [21] or directly blocking caspase activation [38]. Down-regulation of NF-κB could therefore result in a decrease in crucial anti-apoptotic influences both at the mitochondrial and the membrane death receptors levels. Most strikingly, these actions are unique to cimetidine and are not shared by the other H2R antagonists tested. This report, therefore, clearly demonstrates that cimetidine inhibits NF-κB activation, with subsequent down-regulation of the expression of NCAM, and that as a consequence HSG cell proliferation, which requires homophilic NCAM binding, is blocked.

We believe that this series of events provides the mechanism by which cimetidine suppresses the development of salivary gland tumors. Our data also suggest that the anti-tumor activity of cimetidine may rely on inhibition of the transcription factor NF-κB. However, the reason that cimetidine, but not other H2R antagonists, interferes with NF-κB activation remains to be elucidated.

## Conclusion

We investigated the NCAM-associated effect of cimetidine on tumor growth and perineural/neural invasion in salivary gland tumors using an *in vitro *cell culture system and an *in vivo *nude mice cancer model. These experiments clearly demonstrated that cimetidine effectively down-regulates the induction of NCAM by inhibiting the transactivation of NF-κB, which subsequently blocks HSG adhesion to neural cells, and ultimately induces apoptosis in HSG cells and prevents the growth of HSG tumor masses in nude mice. These findings may explain clinical observations by several authors [2,3] that cimetidine improves the survival of patients with malignant tumors. Although malignant glandular tumors are known to be generally resistant to radiation therapy and chemotherapy, the clinical application of cimetidine as an anti-cancer drug might form an integral part of future therapeutic strategies against NCAM-expressing tumors such as adenoid cystic carcinoma. Further studies will be required to identify the signal transduction pathways by which treatment with cimetidine suppresses the growth of glandular tumors and to establish a strategy for cimetidine-based therapy of salivary gland tumors. Although malignant glandular tumors are known to be generally resistant to radiation therapy and chemotherapy, the clinical application of cimetidine as an anti-cancer drug might form an integral part of future therapeutic strategies against NCAM-expressing tumors such as adenoid cystic carcinoma. Further studies will be required to identify the signal transduction pathways by which cimetidine suppresses the growth of glandular tumors and to establish a strategy for cimetidine-based therapy of salivary gland tumors.

## Competing interests

The authors declare that they have no competing interests.

## Authors' contributions

MF performed data analysis and drafted the manuscript. HS and KK supervised all data preprocessing. MF and KK conceived the study.

## Pre-publication history

The pre-publication history for this paper can be accessed here:


